# Association between PT, PT-INR, and in-hospital mortality in critically ill patients with tumors: A retrospective cohort study

**DOI:** 10.3389/fpubh.2023.1036463

**Published:** 2023-03-21

**Authors:** Jia-Dong Liang, Zuo-An Qin, Jin-Hao Yang, Chao-Fen Zhao, Qian-Yong He, Kai Shang, Yu-Xin Li, Xin-Yu Xu, Yan Wang

**Affiliations:** ^1^Department of Breast Oncology, Sun Yat-sen University Cancer Center, Guangzhou, China; ^2^Department of Cardiology, The First People’s Hospital of Changde City, Changde, China; ^3^Department of Nasopharyngeal Carcinoma, Sun Yat-sen University Cancer Center, Guangzhou, China; ^4^Department of Oncology, Affiliated Hospital of Guizhou Medical University, Guiyang, China; ^5^Department of Oncology, Affiliated Cancer Hospital of Guizhou Medical University, Guiyang, China; ^6^Department of Oncology, School of Clinical Medicine, Guizhou Medical University, Guiyang, China

**Keywords:** prothrombin time, PT-INR, in-hospital mortality, cancers, intensive care unit

## Abstract

**Objectives:**

Prothrombin time (PT) and PT-INR are independent predictors of mortality in patients with cancer. The PT and PT-INR of cancer patients are independent predictive variables of mortality. However, whether the PT or PT-INR is related to in-hospital mortality in severely ill patients with tumors remains unknown.

**Design:**

This was a case–control study based on a multicenter public database.

**Settings:**

This study is a secondary analysis of data extracted from 2014 to 2015 from the Electronic Intensive Care Unit Collaborative Research Database.

**Participants:**

The data relevant to seriously ill patients with tumors were obtained from 208 hospitals spread throughout the USA. This research included a total of 200,859 participants. After the samples were screened for patients with combination malignancies and prolonged PT-INR or PT, the remaining 1745 and 1764 participants, respectively, were included in the final data analysis.

**Primary and secondary outcome measures:**

The key evaluation methodology was the PT count and PT-INR, and the main outcome was the in-hospital mortality rate.

**Results:**

After controlling for confounding variables, we found a curvilinear connection between PT-INR and in-hospital mortality (*p* < 0.001), and the inflection point was 2.5. When PT-INR was less than 2.5, an increase in PT-INR was positively associated with in-hospital mortality (OR 1.62, 95% CI 1.24 to 2.13), whereas when PT-INR was greater than 2.5, in-hospital mortality was relatively stable and higher than the baseline before the inflection point. Similarly, our study indicated that the PT exhibited a curvilinear connection with in-hospital mortality. On the left side of the inflection point (PT <22), a rise in the PT was positively linked with in-hospital mortality (OR 1.08, 95% CI 1.04 to 1.13, *p* < 0.001). On the right side of the inflection point, the baseline PT was above 22, and the in-hospital mortality was stable and higher than the PT count in the prior range (OR 1.01, 95% CI 0.97 to 1.04, 0.7056).

**Conclusion:**

Our findings revealed that there is a curved rather than a linear link between the PT or PT-INR and in-hospital mortality in critically ill cancer patients. When these two laboratory results are below the inflection point, comprehensive therapy should be employed to reduce the count; when these two laboratory results are above the inflection point, every effort should be made to reduce the numerical value to a value below the inflection point.

## Background

In clinical labs, PT is the most often used coagulation test to achieve test findings that are adjusted for thromboplastin and the instrument used. The universal acceptance of the PT test, however, has not resulted in an oral anticoagulant dose that is uniform or safe due to the lack of thromboplastin standardization in the PT test as well as the diverse methods used to report results. (1) PT is mathematically transformed to the PT international normalized ratio (PT-INR) for use in monitoring anticoagulant treatment with vitamin K antagonists, such as warfarin. Using the mean normal PT (MNPT) and the international sensitivity index (ISI), the INR is calculated accurately. In addition, manufacturers of reagents and coagulometers have made attempts to harmonize INRs, making INR use more acceptable to influence patient care accurately. (2)

In addition to assessing clinical bleeding risk, PT also serves as a prognostic predictive biomarker for newly diagnosed multiple carcinomas, such as colorectal cancer, renal cancer, multiple myeloma, and hepatocellular carcinoma. (3–6) These studies show that numerical fluctuations in PT may be used as an independent factor to predict relapse-free survival (RFS) and recurrence. Many laboratory test indices, such as platelets and fibrinogen, which can affect the level of PT, have recently been proven to be directly related to the microenvironment in carcinoma or the occurrence and metastasis of tumors. (7)

It is believed that an abnormal PT has significant influence on the deteriorating prognosis of intensive care unit (ICU) patients. An abnormal platelet count, which critically affects the PT count, is one of the most prevalent issues in ICUs. (8) Studies have indicated that abnormal levels of PT are related to an increased incidence of hematological malignancies (9) and an increase in mortality in critically ill individuals. (10)

However, in patients with severe tumors, the relationship between PT, PT-INR and in-hospital mortality remains uncertain. Given the methodological constraints, it is difficult to make a valid conclusion about the association between these parameters and the cutoff values for PT or PT-INR in patients with severe malignancies. Therefore, we performed a retrospective cohort analysis to investigate the association between PT or PT-INR and in-hospital mortality in patients with severe tumors and to evaluate the PT or PT-INR cutoff point that indicates a decreasing risk of mortality to serve as a reference to be used in clinical practice.

## Methods

### Data source and study population

**Figure 1:**
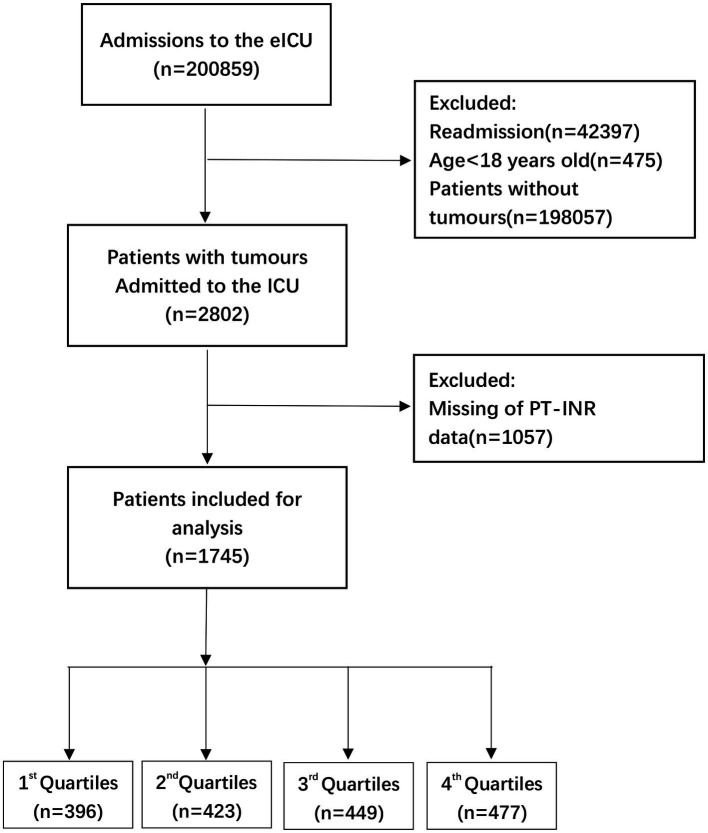
Flow chart of patient selection related to PT-INR. eICU, electronic intensive care unit; ICU, intensive carse unit.

**Figure 2:**
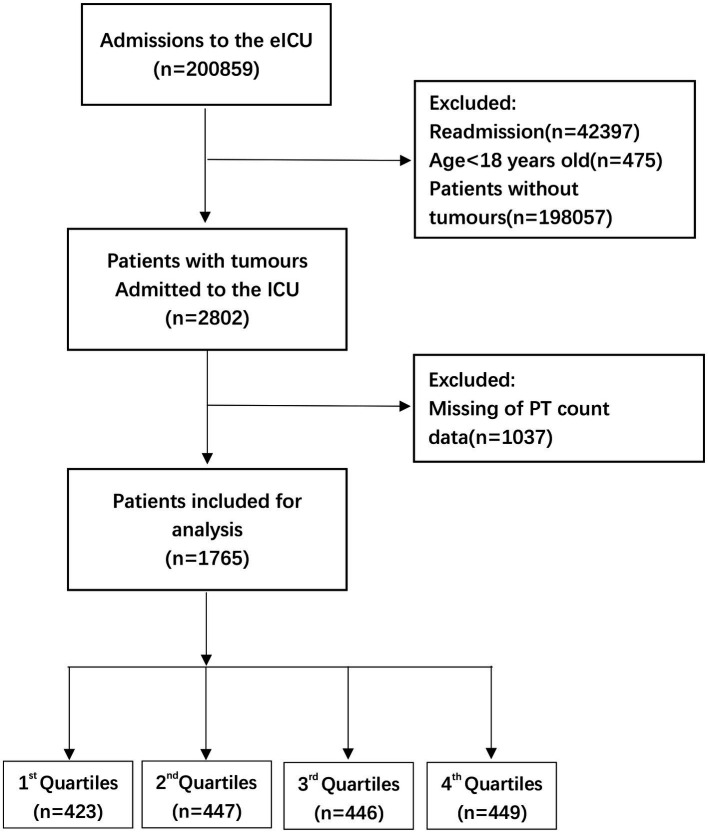
Flow chart of patient selection related to PT count. eICU, electronic intensive care unit; ICU, intensive care unit.

This research used data acquired from the Electronic Intensive Care Unit (eICU) Collaborative Research Database (eICU-CRD) encompassing 200,859 ICU admissions in 208 US hospitals from 2014 to 2015. After finishing the web-based training courses and the Protecting Human Research Participants examination (No. 36208651), we obtained permission to extract data from the eICU-CRD. In this research, the clinical patient information was converted to nontraceable codes to preserve patient privacy. The hospital’s electronic medical records system was mined for information, including physical and medical results, pharmaceutical records, laboratory reports, and imaging data. After completing online training classes and the test on Protecting Human Research Participants (No. 36208651), we were granted authorization to collect data from the eICU-CRD. We analyzed the information included in this public database in depth. (11) The statistics from the eICU website^1^ were analyzed thoroughly in this study. We selected individuals based on the following exclusion criteria: (1) individuals under the age of 18 (*n* = 475); (2) subjects without carcinoma data (*n* = 198,057); (3) readmitted patients (*n* = 42,397); and (4) participants with missing PT-INR or PT values (*n* = 1,057 and *n* = 1,037, respectively). Therefore，there are 1745 and 1765 individuals, respectively, for the final data analysis (see the flowcharts in Figures 1, 2 for more details).

### Study design

The research study was a secondary analysis of a dataset that was generated from a multicenter cohort. In this investigation, both PT and PT-INR were considered independent variables, and both were presented as continuous variables. The outcome variable, in-hospital mortality rate, was recorded as a binary variable, with 1 representing death and 0 representing survival.

### Clinical variables and outcomes

The independent variable in this research was PT-INR or PT. The target dependent variable was in-hospital mortality.

Age is the continuous variable among covariates (years). The categorical variables consisted of sex (male or female), race/ethnicity (African American, Asian, Caucasian, Hispanic, Native American, Other/Unknown), tumor type (hematologic malignancy, chest tumors, skin, muscle and skeletal tumors, gastrointestinal (GI) tumors, head and neck tumors, central nervous system (CNS) tumors, unknown primary tumors, and genitourinary (GU) tumors), cooccurring usage of anticoagulant or antiplatelet medications, mechanical ventilation, and glucocorticoid use.

### Statistics and the absence of data

We accounted for significant variation using ANOVA and χ^2^ tests. Continuous variables are given as the mean and standard deviation. Categorical variables are reported as frequencies or percentages. Our statistical study comprised three primary phases to determine whether either PT or PT-INR was associated with in-hospital mortality in the sampled subjects.

Step 1: Univariate and multivariate logistic regression models with binary variables were developed. We developed three separate models: an unadjusted model (without adjusting for variables), a minimally adjusted model (with only sociodemographic factors modified), and a fully adjusted model (with variables adjusted as reported in Tables 1 and 2).

**Table 1:** Characteristics and outcomes of participants.

PT-INR(ratio)quartile	Q1	Q2	Q3	Q4	*p* value
*N*	396	423	449	477	
Demographics					
Age (years, mean ± sd)	64.93 ± 13.57	65.54 ± 13.53	66.81 ± 13.10	66.99 ± 13.93	0.074
Gender, *n* (%)					0.008
Male	189 (47.73%)	236 (55.79%)	243 (54.12%)	282 (59.12%)	
Female	207 (52.27%)	187 (44.21%)	206 (45.88%)	195 (40.88%)	
Ethnicity, *n* (%)					0.029
African American	25 (6.38%)	34 (8.17%)	57 (12.75%)	45 (9.55%)	
Asian	5 (1.28%)	3 (0.72%)	2 (0.45%)	5 (1.06%)	
Caucasian	296 (75.51%)	289 (69.47%)	286 (63.98%)	336 (71.34%)	
Hispanic	55 (14.03%)	77 (18.51%)	83 (18.57%)	63 (13.38%)	
Native American	4 (1.02%)	2 (0.48%)	3 (0.67%)	4 (0.85%)	
Other/Unknown	7 (1.79%)	11 (2.64%)	16 (3.58%)	18 (3.82%)	
Tumor type					0.447
Hematologic malignancy	51 (12.88%)	55 (13.00%)	60 (13.36%)	64 (13.42%)	
Chest tumors	114 (28.79%)	123 (29.08%)	112 (24.94%)	136 (28.51%)	
Skin, muscle and skeletal tumors	10 (2.53%)	13 (3.07%)	10 (2.23%)	17 (3.56%)	
GI tumors	97 (24.49%)	100 (23.64%)	113 (25.17%)	122 (25.58%)	
Head and neck tumors	37 (9.34%)	37 (8.75%)	51 (11.36%)	46 (9.64%)	
CNS tumors	14 (3.54%)	28 (6.62%)	29 (6.46%)	30 (6.29%)	
Unknown primary tumor	6 (1.52%)	2 (0.47%)	5 (1.11%)	10 (2.10%)	
GU tumors	67 (16.92%)	65 (15.37%)	69 (15.37%)	52 (10.90%)	
Clinical factors					
ARF					<0.001
No	323 (81.57%)	322 (76.12%)	322 (71.71%)	335 (70.23%)	
Yes	73 (18.43%)	101 (23.88%)	127 (28.29%)	142 (29.77%)	
ACS					0.498
No	371 (93.69%)	405 (95.74%)	425 (94.65%)	456 (95.60%)	
Yes	25 (6.31%)	18 (4.26%)	24 (5.35%)	21 (4.40%)	
Coagulopathy					<0.001
No	395 (99.75%)	420 (99.29%)	433 (96.44%)	416 (87.21%)	
Yes	1 (0.25%)	3 (0.71%)	16 (3.56%)	61 (12.79%)	
Antiplatelet drugs					<0.001
No	347 (87.63%)	389 (91.96%)	414 (92.20%)	457 (95.81%)	
Yes	49 (12.37%)	34 (8.04%)	35 (7.80%)	20 (4.19%)	
Anticoagulant drugs					0.102
No	382 (96.46%)	412 (97.40%)	431 (95.99%)	470 (98.53%)	
Yes	14 (3.54%)	11 (2.60%)	18 (4.01%)	7 (1.47%)	
Glucocorticoid					0.147
No	323 (81.57%)	359 (84.87%)	360 (80.18%)	405 (84.91%)	
Yes	73 (18.43%)	64 (15.13%)	89 (19.82%)	72 (15.09%)	
Mechanical ventilation					0.086
No	325 (82.07%)	341 (80.61%)	346 (77.06%)	362 (75.89%)	
Yes	71 (17.93%)	82 (19.39%)	103 (22.94%)	115 (24.11%)	
ICU mortality					<0.001
No	378 (95.45%)	401 (94.80%)	398 (88.64%)	397 (83.23%)	
Yes	18 (4.55%)	22 (5.20%)	51 (11.36%)	80 (16.77%)	

PT-INR: International coagulation ratio; ARF: Acute respiratory failure; ACS: Acute coronary syndrome; ICU: intensive care unit

**Table 2:** Characteristics and outcomes of participants.

PT quartile	Q1	Q2	Q3	Q4	*p* value
*N*	423	447	446	449	
Demographics					
Age (years, mean ± sd)	64.96 ± 13.85	65.82 ± 12.81	66.81 ± 13.75	67.10 ± 13.72	0.080
Gender, *n* (%)					0.069
Male	219 (51.77%)	228 (51.01%)	256 (57.40%)	260 (57.91%)	
Female	204 (48.23%)	219 (48.99%)	190 (42.60%)	189 (42.09%)	
Ethnicity, *n* (%)					<0.001
African American	43 (10.29%)	33 (7.45%)	49 (11.06%)	38 (8.56%)	
Asian	5 (1.20%)	3 (0.68%)	3 (0.68%)	4 (0.90%)	
Caucasian	346 (82.78%)	302 (68.17%)	265 (59.82%)	311 (70.05%)	
Hispanic	23 (5.50%)	90 (20.32%)	101 (22.80%)	67 (15.09%)	
Native American	0 (0.00%)	4 (0.90%)	5 (1.13%)	4 (0.90%)	
Other/Unknown	1 (0.24%)	11 (2.48%)	20 (4.51%)	20 (4.50%)	
Tumor type					<0.001
Hematologic malignancy	57 (13.48%)	59 (13.20%)	58 (13.00%)	59 (13.14%)	
Chest tumors	111 (26.24%)	122 (27.29%)	125 (28.03%)	131 (29.18%)	
Skin, muscle and skeletal tumors	26 (6.15%)	9 (2.01%)	3 (0.67%)	14 (3.12%)	
GI tumors	89 (21.04%)	103 (23.04%)	126 (28.25%)	118 (26.28%)	
Head and neck tumors	53 (12.53%)	43 (9.62%)	39 (8.74%)	39 (8.69%)	
CNS tumors	24 (5.67%)	23 (5.15%)	24 (5.38%)	31 (6.90%)	
Unknown primary	3 (0.71%)	5 (1.12%)	8 (1.79%)	7 (1.56%)	
GU tumors	60 (14.18%)	83 (18.57%)	63 (14.13%)	50 (11.14%)	
Clinical factors					
ARF					<0.001
No	343 (81.09%)	344 (76.96%)	318 (71.30%)	312 (69.49%)	
Yes	80 (18.91%)	103 (23.04%)	128 (28.70%)	137 (30.51%)	
ACS					0.054
No	393 (92.91%)	427 (95.53%)	422 (94.62%)	435 (96.88%)	
Yes	30 (7.09%)	20 (4.47%)	24 (5.38%)	14 (3.12%)	
Coagulopathy					<0.001
No	420 (99.29%)	444 (99.33%)	434 (97.31%)	378 (84.19%)	
Yes	3 (0.71%)	3 (0.67%)	12 (2.69%)	71 (15.81%)	
Antiplatelet drugs					0.001
No	385 (91.02%)	399 (89.26%)	410 (91.93%)	432 (96.21%)	
Yes	38 (8.98%)	48 (10.74%)	36 (8.07%)	17 (3.79%)	
Anticoagulant drugs					0.008
No	413 (97.64%)	431 (96.42%)	426 (95.52%)	445 (99.11%)	
Yes	10 (2.36%)	16 (3.58%)	20 (4.48%)	4 (0.89%)	
Glucocorticoid					<0.001
No	375 (88.65%)	361 (80.76%)	352 (78.92%)	379 (84.41%)	
Yes	48 (11.35%)	86 (19.24%)	94 (21.08%)	70 (15.59%)	
Mechanical ventilation					<0.001
No	372 (87.94%)	353 (78.97%)	327 (73.32%)	337 (75.06%)	
Yes	51 (12.06%)	94 (21.03%)	119 (26.68%)	112 (24.94%)	
ICU mortality					<0.001
No	405 (95.74%)	415 (92.84%)	396 (88.79%)	372 (82.85%)	
Yes	18 (4.26%)	32 (7.16%)	50 (11.21%)	77 (17.15%)	

PT-INR: International coagulation ratio; ARF: Acute respiratory failure; ACS: Acute coronary syndrome; ICU: intensive care unit

Step 2: Given that logistic regression cannot handle the nonlinear connection and that a nonlinear association between log^2^ PT-INR, PT and mortality cannot be ruled out, smooth curve fitting (penalized spline approach) was used to account for nonlinearity. After calculating the inflection point using a recursive approach, we developed a two-piecewise linear regression model on both sides of the inflection point when nonlinearity was discovered. Based on the *p* values for the log likelihood ratio test, we selected the model of best fit (linear regression vs. two-piecewise linear regression). In this stage, the R packages ggplot2, nlme, and mgcv were used.

Step 3: To guarantee the reliability of the data analysis, a sensitivity analysis was conducted. The objective was to validate the findings obtained using continuous PT-INR and PT for variables. This research used the “P for trend” statistic for sensitivity analysis. To guarantee the robustness of data processing, we changed the log^2^ PT-INR and PT values into quartile-based categorical variables for sensitivity analysis to ensure the validity and reliability of log^2^ PT-INR and PT as continuous variables.

**Figure 3:**
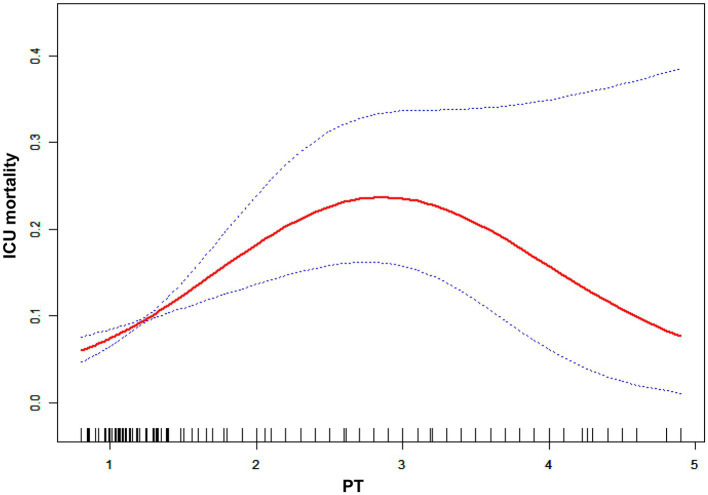
Nonlinear relationship between the PT-INR and in-hospital mortality.

**Figure 4:**
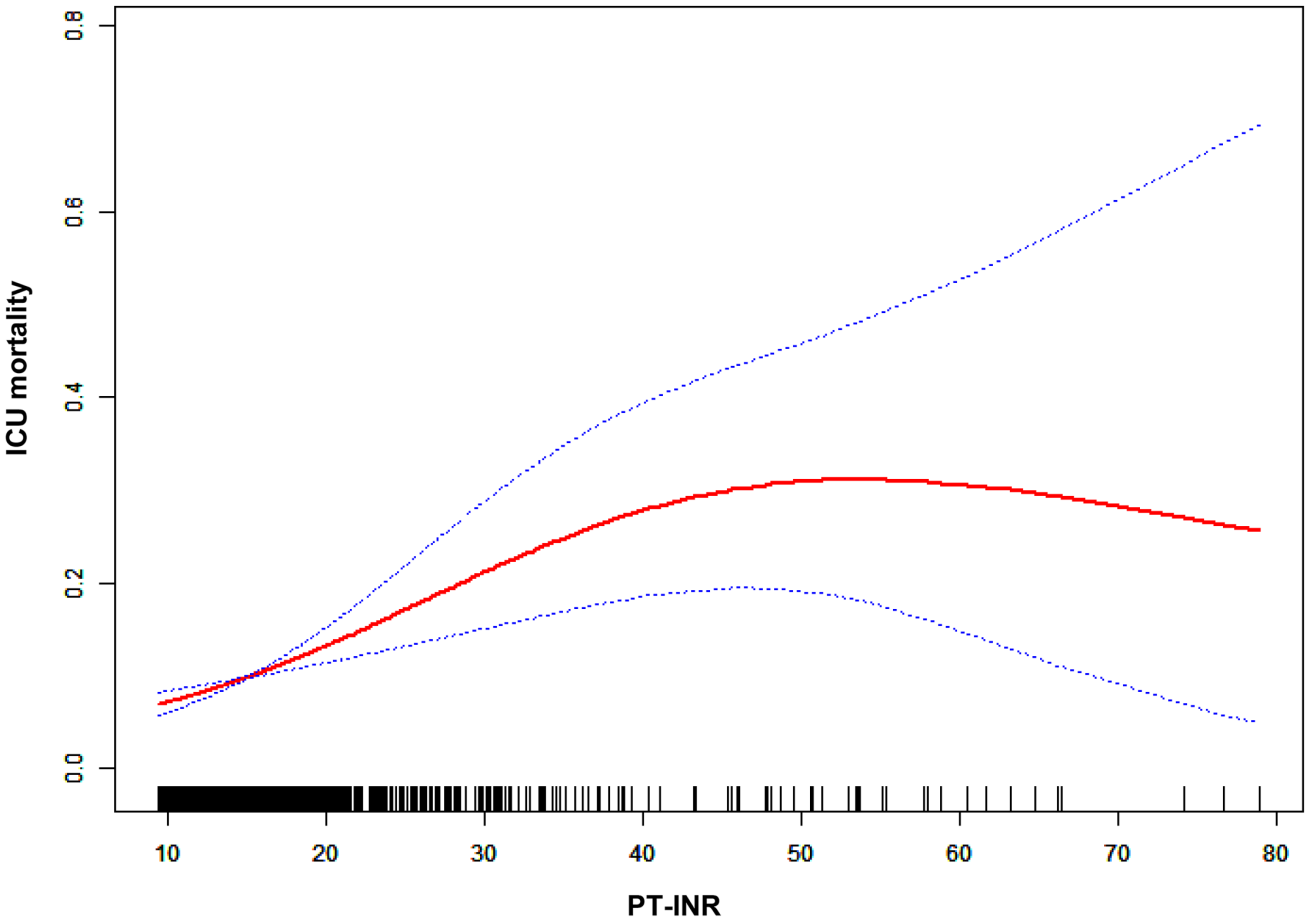
Nonlinear relationship between the PT count and in-hospital mortality.

The percentages of missing data of PT-INR and PT analysis are 37.72 and 37.01%, respectively (see the flowcharts in Figures 1, 2 for more details). In order to exclude the impact of missing values on the overall results, multiple imputation was used for missing variables. In online Supplementary Table 1-1 and Table 2, we reported missing data for each variable for the 1745 and 1765 participants, respectively, in the analytic sample. Our objective is to minimize potential deviations and increase predictive significance. Covariates without data were omitted from the data analysis. (12) We produced five sets of imputation data using multiple imputation with a Mice software package and performed sensitivity analysis on the data prior to imputation. (13) We observed that the data were almost identical before and after imputation, and the six curves exhibited a similar trend. Details can be found in the online Supplementary Table 1-1, Table 2, and Figures 3, 4.

The analyses were conducted using the statistical software programs R (http://www.R-project.org, The R Foundation) and EmpowerStats (http://www. empowerstats.com, X&Y Solutions, Inc., Boston, MA). *p* values (two-sided) less than 0.05 were deemed statistically significant.

## Results

### Baseline characteristics of participants

The distributions of baseline characteristics related to PT-INR and PT are given in Tables 1 and 2, respectively. Table 1 displays the weighted distributions of sociodemographic traits and other concomitant variables in the chosen eICU population from 2014 to 2015. The mean patient ages were 66.12 ± 14.89 and 66.19 ± 14.19 years and the proportion of males were 54 and 55% for patients related to PT-INR and PT, respectively. The ICU mortality rates related to PT-INR and PT were 14.24 and 13.69%, respectively. There were no statistically significant differences between quartiles of PT-INR and age, tumor type, ACS, anticoagulant drugs, glucocorticoids, or mechanical ventilation. There were no substantial differences in age, sex or ACS among individuals in various quartiles of PT (all *p* > 0.05).

According to Table 1, 18 individuals in the first quartile for PT-INR died, in addition to 22 participants in the second quartile, 51 patients in the third quartile, and 80 patients in the fourth quartile. The majority of the terminally ill cancer patients were Caucasian, and just a few were Asian or Native American. The number of patients with GU cancers in the fourth quartile was fewer than that in the three other quartiles. In addition, we found that the number of participants in the first quartile was fewer than the number of patients in the other three quartiles, while the number of patients under mechanical ventilation was the reverse. As stated in the table, the most prevalent kinds of cancers were hematologic malignancies, chest tumors, GI tumors, head and neck tumors, and GU tumors.

As shown in Table 2, 18 patients in the first quartile for PT perished, in addition to 32 in the second quartile, 50 in the third, and 77 in the fourth. The bulk of terminally ill cancer patients were Caucasian and African American, and just a few were Asian, Hispanic, or Native American. Among the different PT quartiles, the percentage of patients with skin, muscle, and skeletal tumors in the first quartile was significantly higher than that in the other quartiles.

Patients with GU cancer in the second quartile outnumbered those in the other three quartiles. Patients with head and neck tumors in the first quartile surpass those in the next three quartiles. In the fourth quartile, the use of antiplatelet medications was greater than that in the other quartiles, although the use of anticoagulant drugs and the likelihood of coagulopathy in patients were the opposite.

### Relationship between PT-INR (ratio) and in-hospital mortality

In Table 3, we outlined the magnitudes of the correlation between PT-INR and in-hospital mortality. Model 1 (the crude model) was not altered. This model revealed a correlation between PT-INR and in-hospital mortality (OR 1.72, 95% CI 1.38 to 2.15). Using sensitivity analysis, the integrity of these data was verified. In Model 2 (minimally adjusted model), the association between PT-INR and in-hospital mortality remained positive after adjusting for sociodemographic characteristics (age, sex, and ethnicity; OR 1.74, 95% CI 1.39 to 2.18). Model 3 (the model with all modifications) had comparable results (OR 1.62, 95% CI 1.24 to 2.13). Figure 3 and Table 4 illustrate the nonlinear relationship between the PT-INR and in-hospital mortality. After adjusting for demographic data, malignant tumors, treatments, and comorbidities, the recursive technique revealed a curvilinear relationship between PT-INR and in-hospital mortality (*p* = 0.0005). The inflection point was 2.5. On the left side of the inflection point (PT-INR = 2.5), an increase in PT-INR was positively associated with the hospital (OR 2.64, 95% CI 1.24 to 2.13, *p* < 0.0005). On the right side of the inflection point (PT-INR >2.5), the baseline PT-INR was over 2.5, and in-hospital mortality was greater than the PT-INR in prior ranges. The data distribution is typically skewed, and there are few patients whose PT-INR result is over 2.5; thus, it is straightforward to determine that only 5% of the participants had a PT-INR result greater than 2.4 and only 1% had a PT-INR of more than 4.9, which is shown in detail in Figure 3 and Figure S1. Given the reasons mentioned above, the trend in Figure 3 did not ideally depict the real data situation as in Figure 4, which is related to the PT model. After using multiple imputation, which can maximize statistical power and minimize the bias that might occur for covariates with missing data, it is evident from Supplementary Figure 2-1 and Supplementary Table 1-1 that the data trend is closer to our ideal model, growing linearly before the reflection point and reaching a rather steady peak thereafter.

**Table 3:** The results of multivariate analysis using nonadjusted and adjusted Cox regression models.

Variable	Crude model (OR, 95%CI, *p*)	Minimally adjusted model (OR, 95%CI, *p*)	Fully adjusted model (OR, 95%CI, *p*)
PT-INR(ratio)	1.72 (1.38, 2.15) <0.0001	1.74 (1.39, 2.18) <0.0001	1.62 (1.24, 2.13) 0.0005
PT-INR(ratio)quartile			
Q1	Ref	Ref	Ref
Q2	1.15 (0.61, 2.18) 0.6638	1.18 (0.62, 2.23) 0.6143	1.06 (0.54, 2.09) 0.8588
Q3	2.69 (1.54, 4.69) 0.0005	2.58 (1.48, 4.52) 0.0009	2.26 (1.24, 4.11) 0.0075
Q4	4.23 (2.49, 7.19) <0.0001	4.31 (2.53, 7.35) <0.0001	3.71 (2.08, 6.63) <0.0001
*p* for trend	<0.0001	<0.0001	<0.0001

Crude model: No covariables were modified. Minimally adjusted model: Only sociodemographic variables were modified. Fully adjusted model: All covariates presented in Table 1 were modified. OR: Odds ratio; CI: confidence interval; Ref: reference.

**Table 4:** Nonlinearity of the PT-INR (ratio) on ICU mortality.

Outcome	Effect size (95%CI) *p* value
Model of best fit using binary logistic regression	1.62 (1.24, 2.13) 0.0005
Model of best fit using two-piecewise linear model	
Inflection point	2.5
< 2.5	2.64 (1.64, 4.23) <0.0001
> 2.5	0.65 (0.28, 1.53) 0.3268
*p* for Log likelihood ratio test	0.016

All results shown in Table 6 were calculated by preimputation data. The adjustment strategy was the same as that for the fully adjusted model.

A logistic regression model revealed that on the left side of the inflection point, the chance of in-hospital mortality rose by 164% with each increment of 1 in the PT-INR (OR 2.64, 95% CI 1.64 to 4.23, *p* < 0.0001). In addition, on the right side of the inflection point, an increase in PT-INR had no effect on the likelihood of in-hospital mortality (OR 0.65, 95% CI 0.28, 1.53, *p* = 0.3268). PT-INR demonstrated a nonlinear relationship with in-hospital mortality.

### Relationship between PT and in-hospital mortality

The impact sizes of the connection between PT and in-hospital mortality are shown in Table 5. Model 1 (the crude model) was not modified. This model demonstrated a positive association between PT count and in-hospital mortality (OR 1.04, 95% CI 1.02 to 1.06). The veracity of these data was confirmed by sensitivity analysis. After correcting for sociodemographic factors (age, sex, and ethnicity) in Model 2 (minimally adjusted model), the link between PT and in-hospital mortality remained positive (OR 1.04, 95% CI 1.03 to 1.06). Similar findings were seen with Model 3 (the model with all adjustments made; OR 1.04, 95% CI 1.02 to 1.06). Figure 4 and Table 6 depict the findings of the nonlinearity of the connection between PT and in-hospital mortality. The recursive method, following correction for demographic data, malignant tumors, therapies and comorbidities, indicated that the PT count exhibited a curvilinear connection with in-hospital mortality (*p* < 0.001). The inflection point was 22. On the left side of the inflection point (PT <22), a rise in the PT count was positively linked with in-hospital mortality (OR 1.08, 95% CI 1.04 to 1.13, *p* < 0.0001). On the right side of the inflection point, the baseline PT was higher, and the in-hospital mortality was greater than the PT count in prior ranges (OR 1.01, 95% CI 0.97 to 1.04, *p* = 0.7056). Multiple imputation was used to increase statistical power and eliminate potential bias, and it is clear that the postimputation trend of the data closely resembles that of the preimputation data, as seen in Supplementary Figure 2-2 and Supplementary Table 1-2.

**Table 5:** The results of multivariate analysis using nonadjusted and adjusted Cox regression models.

Variable	Crude model (OR, 95%CI, *p*)	Minimally adjusted model (OR, 95%CI, *p*)	Fully adjusted model (OR, 95%CI, *p*)
PT	1.04 (1.02, 1.06) <0.0001	1.04 (1.03, 1.06) <0.0001	1.04 (1.02, 1.06) <0.0001
PT quartile			
Q1	Ref	Ref	Ref
Q2	1.73 (0.96, 3.14) 0.0688	1.79 (0.99, 3.24) 0.0555	1.61 (0.86, 3.03) 0.1375
Q3	2.84 (1.63, 4.95) 0.0002	2.78 (1.59, 4.87) 0.0004	2.20 (1.21, 4.03) 0.0103
Q4	4.66 (2.74, 7.93) <0.0001	4.82 (2.82, 8.22) <0.0001	3.95 (2.20, 7.10) <0.0001
*p* for trend	<0.0001	<0.0001	<0.0001

Crude model: No covariables were modified. Minimally adjusted model: Only sociodemographic variables were modified. Fully adjusted model: All covariates presented in Table 2 were modified. OR: Odds ratio; CI: confidence interval; Ref: reference.

**Table 6:** Nonlinearity of PT on ICU mortality.

Outcome	Effect size (95%CI) *p* value
Model of best fit using binary logistic regression	1.04 (1.02, 1.06) <0.0001
Model of best fit using two-piecewise linear model	
Inflection point	22
<22	1.15 (1.09, 1.22) <0.0001
>22	1.00 (0.97, 1.03) 0.8596
*p* for Log likelihood ratio test	<0.001

All results shown in Table 6 were calculated by preimputation data. The adjustment strategy was the same as that for the fully adjusted model.

A logistic regression model revealed that on the left side of the inflection point, the chance of in-hospital mortality rose by 8% with each increment of 1 in PT (OR 1.08, 95% CI 1.04 to 1.13). Additionally, on the right side of the inflection point, a rise in PT did not impact the probability of in-hospital mortality. The PT revealed a curvilinear connection with in-hospital mortality.

We tried for an interaction between PT-INR, PT and other model variables, but none was detected. Please refer to Tables 4 and 6 for details.

## Discussion

PT-INR and PT abnormalities are widespread in the ICU, and our primary objective was to examine the association between the PT-INR or PT and in-hospital mortality among severely ill cancer patients. In this investigation, after adjusting for demographic characteristics, neoplasm type, comorbidities, and therapies, we found a curvilinear association between the PT-INR, PT and in-hospital mortality of terminally ill cancer patients. Nonlinearity analysis revealed that when the PT-INR was less than 2.5, an increase in PT-INR was substantially linked to an increase in the probability of in-hospital mortality; the same was true when PT was less than 22. To the best of our knowledge, this is the first study to reveal a curvilinear association between PT-INR, PT and in-hospital mortality in severely ill cancer patients. This finding indicates that patient mortality can be lowered if we carefully manage the PT-INR and PT to less than 2.5 and 22, respectively, and try to keep it as low as possible within this range. These data will be helpful for providing a more comprehensive insight into the relationship between PT-INR, PT and the prognosis of severely ill cancer patients, with the intention of minimizing adverse events by preserving the prescribed range for the PT-INR and PT level of critically ill individuals with tumors.

Using Cox regression analysis, Nobuhisa et al. performed groundbreaking research demonstrating that a PT-INR over 1.6 on postoperative Day 5 is a reliable indicator of death and serious complications following living-donor liver transplantation, which is an effective treatment for liver cancer. (14) Rui et al. revealed that in ICU patients, PT levels were related to an increased risk of 28-day and overall death. (15) In addition, extended PT-INR was related to shortened recurrence-free survival and overall survival, rendering it a predictive factor of overall survival in patients with pancreatic ductal adenocarcinoma who had undergone curative resection. (16) Our findings are somewhat compatible with the aforementioned findings; specifically, a greater PT-INR was shown to be related to an increased risk of in-hospital mortality in critically ill patients with tumors who were treated in the ICU. In contrast to previous investigations, our study cohort consisted of severely ill individuals with tumors, and the previous research findings cannot be generalized to this patient group, as it consists of diverse kinds of malignancies rather than specific malignancies in previous studies. Previous research solely explored a linear association between PT-INR and mortality, which is an oversimplification of the complexity of biological processes. In contrast, in our study, we examined the possibility of a nonlinear relationship and discovered that when PT-INR is less than 2.5, an increase in PT-INR is positively associated with in-hospital mortality, whereas when PT-INR is greater than 2.5, in-hospital mortality is relatively stable and higher than in the prior range. In other words, the mortality rate will not grow indefinitely as the PT-INR rises, and it will peak when the PT-INR is more than 2.5.

In clinical practice, the significance of PT is comparable to that of PT-INR, and an increase in PT correlates with an increase in in-hospital mortality. Bayir et al. performed a study on trauma patients, and PT count was associated with increased mortality in the intensive care unit. (17) Wang et al. performed a pathbreaking study demonstrating that an elevated PT level was an excellent predictor of overall survival and recurrence-free survival in patients with cholangiocarcinoma, regardless of age, tumor type, or TNM stage. In the group with low PT levels, the mean overall survival and recurrence-free survival were higher than in the group with high PT levels. There was a very significant correlation between elevated PT levels and decreased overall survival as well as recurrence-free survival. (18) By utilizing univariable and multivariate logistic regression analysis, longer PT are found to be related to an increased risk of in-hospital mortality in patients with lung cancer. (19) In addition, in contrast to prior studies, our study cohort comprised very ill persons with tumors, and past research results cannot be extended to our patient population, which consisted of several types of malignancies as opposed to one. The above studies only investigated the relationship between PT and mortality in the ICU or a linear relationship between PT and mortality in patients with cancers, which is a limited view of the complexity of the processes in the human body.

In contrast, in our study, we assessed whether there was a nonlinear relationship and revealed a curvilinear association when the PT was less than 22. The ICU mortality rate for cancer patients will increase as the PT rises. When PT was over 22, the related mortality in the ICU peaked level, and the connection between the PT and the probability of death was no longer statistically significant once the PT exceeded 22. The ICU mortality rate reached its peak when the PT exceeded 22, and the relationship between the PT and mortality ceased to be statistically significant once the PT count exceeded 22.

There are many reasons for PT-INR or PT count abnormalities in tumor patients in the ICU, and multifactorial mechanisms often act simultaneously. The primary influencing factors involve congenital coagulation factor abnormalities, (20) vitamin K deficiency, (21) severe liver disease, (22) disseminated intravascular coagulation, (23) and various mechanisms of thrombocytopenia, including bone marrow cancer, (24) autoimmune disease, (25) platelet consumption, (26) and bone marrow suppression related to radiation and chemotherapy. (27) Among ICU patients with malignancies, the aforementioned mechanisms may operate in conjunction to enhance the in-hospital mortality. According to studies conducted by Geng et al., (4) PT has a crucial role in determining the prognosis of ICU patients with tumors. An increase in PT and PT-INR is associated with the prevention of tumor metastasis, (28) while a drop in PT and PT-INR heightens the risk of hypercoagulability, which may easily result in myocardial infarction or cerebral thrombosis. (29, 30) The research stated above demonstrated that a rise in PT and PT-INR in critically ill cancer patients in the ICU was related to an increase in mortality. In our research, these patients complied with the above criteria and there was a trend in how mortality changed with increasing PT and PT-INR. This discovery is beneficial for guiding early therapeutic therapies and clinical practice for patients who are at risk, thereby extending their lives.

The large sample size from the eICU-CRD is a key benefit of our investigation, as it significantly enhances the statistical power and reliability of the secondary data analysis. Additionally, this is the first study to demonstrate a curvilinear, as opposed to a linear, association between the PT, PT-INR and in-hospital mortality in critically ill cancer patients. Lastly, these simple-to-obtain laboratory test results, PT and PT-INR were utilized as markers for predicting the prognosis of malignancy. Using these markers in clinical practice should be advocated, given their clinical and practical value. Because of these benefits, the conclusions we reach under challenging circumstances are more valuable and meaningful.

Our study inevitably had its own shortcomings. First, since this study was retrospective, it was susceptible to the inherent limitations of a retrospective design. Second, while we used multivariate logistic regression to compensate for possible confounding variables, several potential confounding factors were omitted from the study, resulting in biased findings. Third, these patients’ laboratory test results fluctuate from laboratory to laboratory owing to demographic variables, blood testing procedures, ethnic blood characteristics and the duration of data collection. Finally, this study is a secondary data-mining study based on eICU-CRD, a public database of freely available clinical data for researchers worldwide. The sampling time of PT or PT-INR, and the dosage of warfarin or other DOACs are absent in this database. In future study, we want to investigate the association between the sample time, the dose of warfarin and DOACs, and the final blood test result.

## Conclusion

Our findings revealed that there is a curved rather than a linear link between PT, PT-INR and in-hospital mortality in critically ill cancer patients. In critically ill patients with tumors, in-hospital mortality increases linearly when PT-INR is less than 2.5 or PT is less than 22. However, once it reaches the inflection point, mortality will peak, which is relatively stable and higher than the range before the inflection point. This finding implies that when these two laboratory values are below the inflection point, comprehensive treatment should be used to lower the count, hence minimizing mortality. Moreover, when these two laboratory results are above the inflection point, we should make every effort to lower the numerical value below the inflection point; otherwise, our therapeutic treatment has no positive impact on the prognosis of cancer patients.

## Data availability statement

The original contributions presented in the study are included in the article/Supplementary material, further inquiries can be directed to the corresponding author.

## Ethics statement

The studies involving human participants were reviewed and approved by Massachusetts Institute of Technology Affiliates. The patients/participants provided their written informed consent to participate in this study.

## Author contributions

J-DL, C-FZ, and YW conceived the idea and contributed to the drafting of the manuscript. Z-AQ obtained permission to use eICU database. J-HY and Q-YH extracted the data. J-DL, KS, and J-HY performed the analysis. Y-XL and X-YX contributed to the material preparation. J-DL, J-HY, and C-FZ helped to edit pictures. YW contributed to the study conception and revision of the manuscript. J-DL and YW approved the final version of the submitted manuscript, and are responsible for the overall content as guarantor. All authors contributed to the article and approved the submitted version.

## Conflict of interest

The authors declare that the research was conducted in the absence of any commercial or financial relationships that could be construed as a potential conflict of interest.

## Publisher’s note

All claims expressed in this article are solely those of the authors and do not necessarily represent those of their affiliated organizations, or those of the publisher, the editors and the reviewers. Any product that may be evaluated in this article, or claim that may be made by its manufacturer, is not guaranteed or endorsed by the publisher.

## Footnotes

^1^ https://eicu-crd.mit.edu/
