# Supplementary figures and images for: Association between PT, PT-INR, and in-hospital mortality in critically ill patients with tumors: A retrospective cohort study
Source: Front Public Health. 2023 Mar 21;11:1036463. doi: 10.3389/fpubh.2023.1036463 (PMC10070679; doi:10.3389/fpubh.2023.1036463)

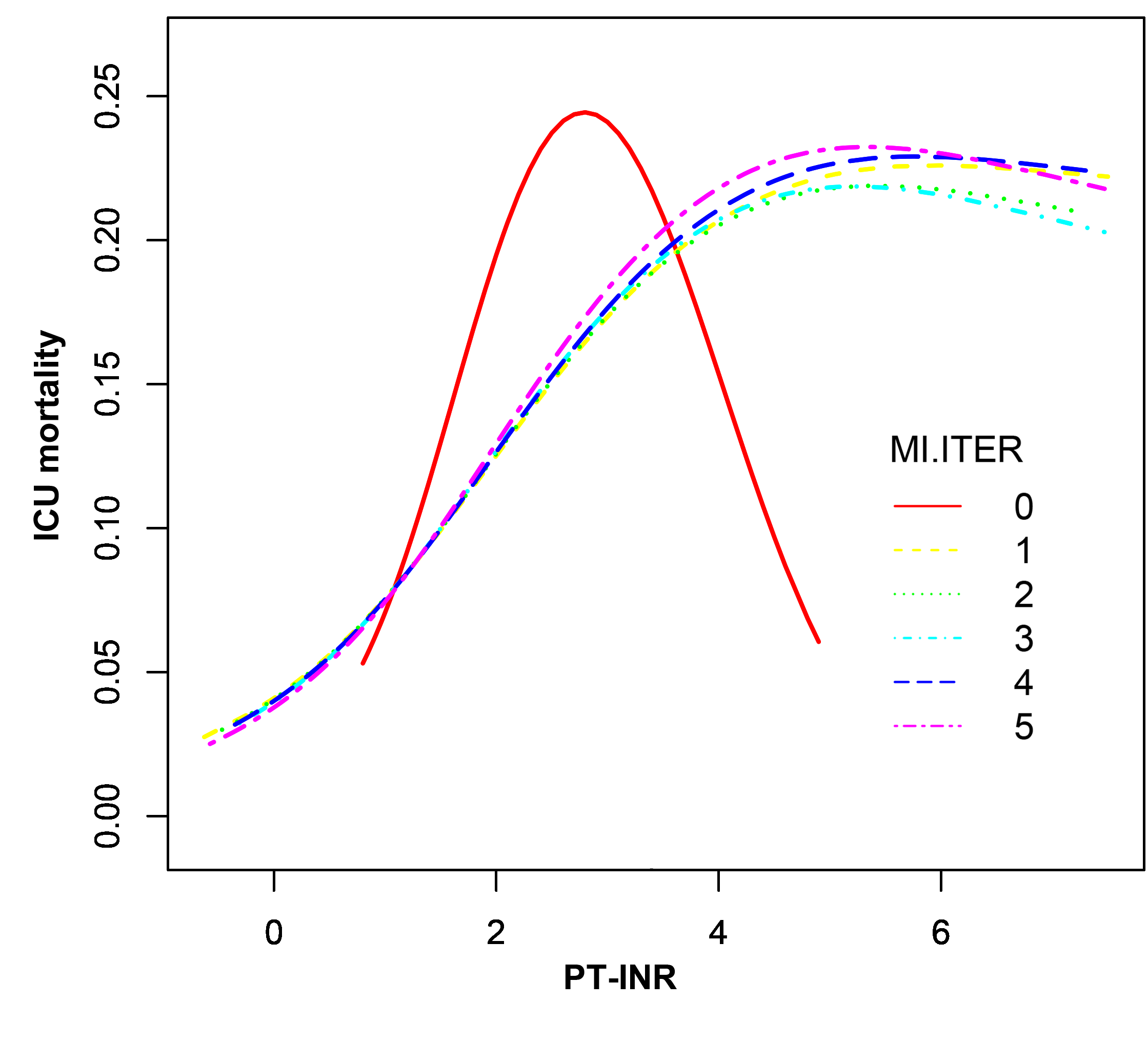

Supplement: Supplementary file 2 [file Image_2.TIF]

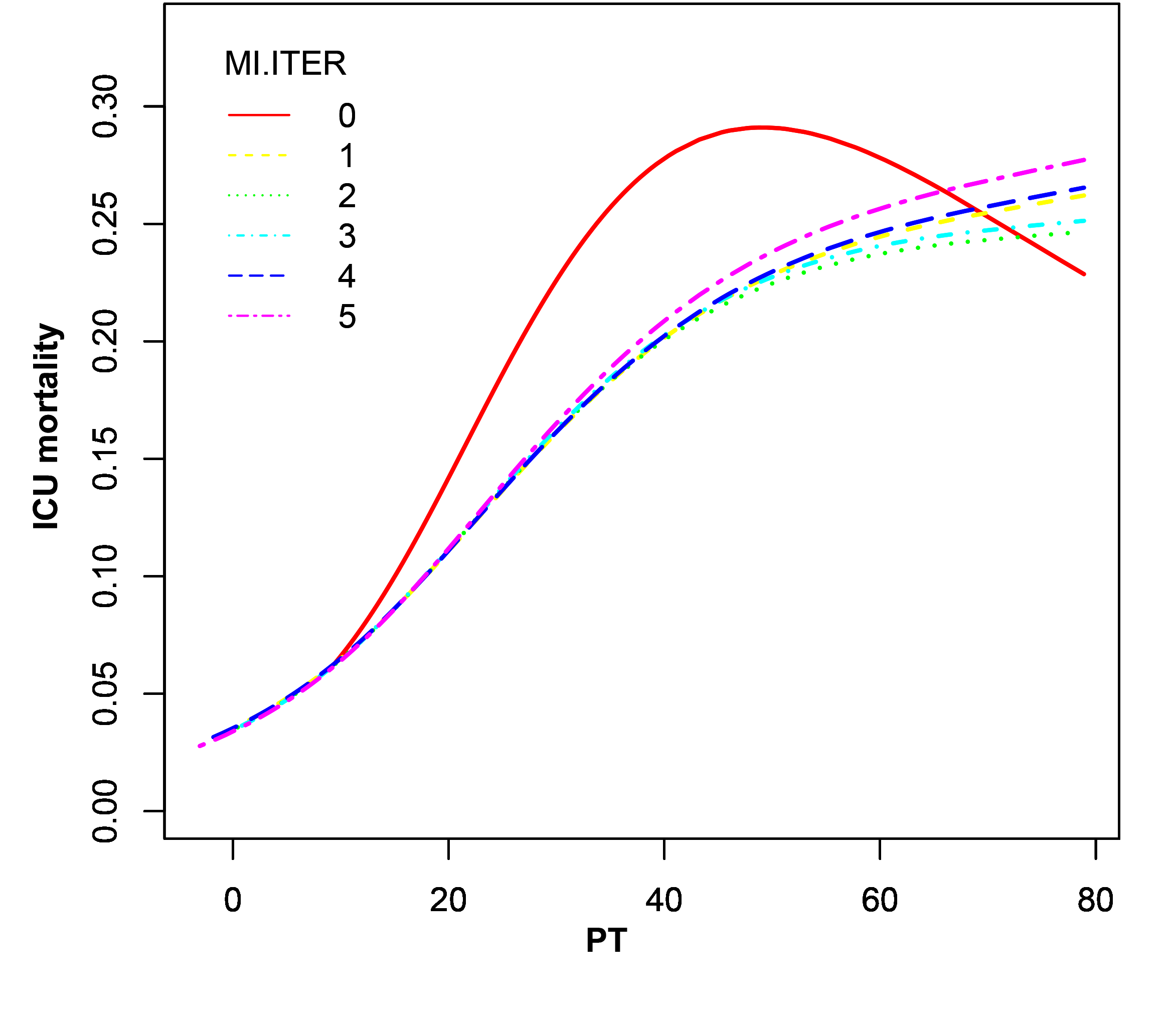

Supplement: Supplementary file 3 [file Image_3.TIF]
