# Supplementary material for: Association between PT, PT-INR, and in-hospital mortality in critically ill patients with tumors: A retrospective cohort study
Source: Front Public Health. 2023 Mar 21;11:1036463. doi: 10.3389/fpubh.2023.1036463 (PMC10070679; doi:10.3389/fpubh.2023.1036463)
Supplement: Supplementary file 4 [file Table_1.DOCX]

|  | Pre-imputation  (OR,95% CI) | Pro-imputation1  (OR, 95% CI) | Pro-imputation2  (OR, 95% CI) | Pro-imputation3  (OR, 95% CI) | Pro-imputation4  (OR, 95% CI) | Pro-imputation5  (OR, 95% CI) |
| --- | --- | --- | --- | --- | --- | --- |
| Model fit using binary logistic regression | 1.70(1.30,2.21) | 1.42 (1.20,1.68) | 1.41 (1.19,1.68) | 1.42 (1.20,1.68) | 1.44 (1.21,1.71) | 1.46 (1.24,1.73) |
| Model fit using two-piecewise linear model |  |  |  |  |  |  |
| Inflection points of the PT-INR | 2.5 | 2.5 | 2.5 | 2.5 | 2.5 | 2.5 |
| ＜2.5 | 2.82 (1.77,4.51) | 1.86 (1.36,2.56) | 1.85 (1.35,2.53) | 1.88 (1.37,2.59) | 1.89 (1.38,2.59) | 1.98 (1.45,2.71) |
| ＞2.5 | 0.67 (0.29,1.53) | 1.07 (0.76,1.50) | 1.07 (0.76,1.50) | 1.06 (0.75,1.49) | 1.08 (0.77,1.51) | 1.07 (0.77,1.49) |
| P for log likelihood ratio test | 0.011 | 0.044 | 0.049 | 0.038 | 0.042 | 0.024 |

Supplement table 1-1: Nonlinearity among pre- and pro-imputation data PT-INR count vs. in-hospital mortality)
