# Supplementary material for: Association between PT, PT-INR, and in-hospital mortality in critically ill patients with tumors: A retrospective cohort study
Source: Front Public Health. 2023 Mar 21;11:1036463. doi: 10.3389/fpubh.2023.1036463 (PMC10070679; doi:10.3389/fpubh.2023.1036463)
Supplement: Supplementary file 5 [file Table_2.DOCX]

|  | Pre-imputation  (OR,95% CI) | Pro-imputation1  (OR, 95% CI) | Pro-imputation2  (OR, 95% CI) | Pro-imputation3  (OR, 95% CI) | Pro-imputation4  (OR, 95% CI) | Pro-imputation5  (OR, 95% CI) |
| --- | --- | --- | --- | --- | --- | --- |
| Model fit using binary logistic regression | 1.04 (1.02,1.06) | 1.04 (1.02,1.05) | 1.04 (1.02,1.05) | 1.04 (1.02,1.05) | 1.04 (1.02,1.05) | 1.04 (1.02,1.06) |
| Model fit using two-piecewise linear model |  |  |  |  |  |  |
| Inflection points of the PT count | 22 | 22 | 22 | 22 | 22 | 22 |
| ＜2.5 | 1.15 (1.09,1.22) | 1.08 (1.04,1.12) | 1.08 (1.04,1.13) | 1.08 (1.04,1.12) | 1.08 (1.04,1.12) | 1.08 (1.04,1.12) |
| ＞2.5 | 1.00 (0.97,1.03) | 1.01 (0.99,1.04) | 1.01 (0.99,1.04) | 1.01 (0.99,1.04) | 1.02 (0.99,1.04) | 1.02 (1.00,1.05) |
| P for log likelihood ratio test | <0.001 | 0.022 | 0.011 | 0.019 | 0.029 | 0.048 |

Supplement table 1-2: Nonlinearity among pre- and pro-imputation data PT count vs. in-hospital mortality)
